# DWI scrolling artery sign for the diagnosis of giant cell arteritis: a pattern recognition approach

**DOI:** 10.1136/rmdopen-2023-003652

**Published:** 2024-03-22

**Authors:** Luca Seitz, Susana Bucher, Lukas Bütikofer, Britta Maurer, Harald M Bonel, Fabian Lötscher, Pascal Seitz

**Affiliations:** 1 Department of Rheumatology and Immunology, Inselspital, University Hospital Bern, University of Bern, Bern, Switzerland; 2 CTU Bern, University of Bern, Bern, Switzerland; 3 Department of Diagnostic, Interventional and Pediatric Radiology, Inselspital, University Hospital Bern, University of Bern, Bern, Switzerland; 4 Campusradiologie, Lindenhof Group, Bern, Switzerland

**Keywords:** Magnetic Resonance Imaging, Giant Cell Arteritis, Vasculitis

## Abstract

**Objectives:**

To investigate the diagnostic accuracy of a pattern recognition approach for the evaluation of MRI scans of the head with diffusion-weighted imaging (DWI) in suspected giant cell arteritis (GCA).

**Methods:**

Retrospectively, 156 patients with suspected GCA were included. The ‘DWI-Scrolling-Artery-Sign’ (DSAS) was defined as hyperintense DWI signals in the cranial subcutaneous tissue that gives the impression of a blood vessel when scrolling through a stack of images. The DSAS was rated by experts and a novice in four regions (frontotemporal and occipital, bilaterally). The temporal, occipital and posterior auricular arteries were assessed in the T1-weighted black-blood sequence (T1-BB). The diagnostic reference was the clinical diagnosis after ≥6 months of follow-up.

**Results:**

The population consisted of 87 patients with and 69 without GCA; median age was 71 years and 59% were women. The DSAS showed a sensitivity of 73.6% and specificity of 94.2% (experts) and 59.8% and 95.7% (novice), respectively. Agreement between DSAS and T1-BB was 80% for the region level (499/624; kappa(κ)=0.59) and 86.5% for the patient level (135/156; κ=0.73). Inter-reader agreement was 95% (19/20; κ=0.90) for DSAS on the patient level and 91.3% (73/80; κ=0.81) on the region level for experts. For expert versus novice, inter-reader agreement for DSAS was 87.8% on the patient level (137/156; κ=0.75) and 91.2% on the region level (569/624; κ=0.77).

**Conclusions:**

The DSAS can be assessed in less than 1 min and has a good diagnostic accuracy and reliability for the diagnosis of GCA. The DSAS can be used immediately in clinical practice.

**WHAT IS ALREADY KNOWN ON THIS TOPIC**

- The T1-black-blood MRI sequence has very good diagnostic accuracy for giant cell arteritis (GCA) but, unlike the diffusion-weighted imaging (DWI) sequence, is not part of a standard head MRI protocol. Segmental DWI scoring of the superficial cranial arteries showed good diagnostic accuracy for suspected GCA.

**WHAT THIS STUDY ADDS**

- The DWI-Scrolling-Artery-Sign shows good diagnostic accuracy and reliability for the diagnosis of GCA, comparable to more time-consuming segmental DWI assessment methods and the T1-black-blood sequence. It can be assessed in less than a minute and can be used by non-experts.

**HOW THIS STUDY MIGHT AFFECT RESEARCH, PRACTICE OR POLICY**

- The DWI-Scrolling-Artery-Sign can be used immediately in clinical practice worldwide and may enable earlier diagnosis of GCA. The DWI-Scrolling-Artery-Sign allows screening for GCA during scanning and can be used in a fast-track approach when GCA is suspected.

## Introduction

Giant cell arteritis (GCA) usually affects the temporal arteries (TAs) and other superficial cranial arteries (SCAs).^1^ Early diagnosis and treatment are important.^2 3^ Confirmation of diagnosis by imaging and/or biopsy is advised.^4–6^ For MRI of SCAs, a post-contrast, high-resolution, fat-suppressed T1-weighted black-blood sequence (T1-BB) on a 3-Tesla scanner is recommended.^4 6^ The T1-BB has certain limitations: long acquisition time; limited availability; requirement for contrast agents.^4 7^ In addition, a T1-BB is not part of a standard MRI protocol of the head and is often not performed because the requesting physician did not mention GCA as a possible differential diagnosis, for example, in the case of headache.

In contrast, diffusion-weighted imaging (DWI) is part of almost every MRI protocol of the head, does not require contrast agents, has a short acquisition time and is essential for stroke imaging and, therefore, available on every MRI scanner.^8^ DWI is based on the detection of random Brownian motion of water molecules. Highly cellular tissue, for example, tissue with inflammatory infiltrates or with cellular oedema, exhibits restricted diffusion. On DWI images, such areas appear as relatively hyperintense regions compared with the surrounding tissue. This is more pronounced on images with high diffusion weighting, characterised by a high b-value (eg, b1000).^9–12^ Previous reports have found SCAs or the aorta with vasculitis to appear hyperintense with DWI.^13–17^ We have confirmed these findings in a recent study, where DWI showed a diagnostic accuracy comparable to the T1-BB in patients with suspected GCA.^18^ To apply the vasculitis grading scheme, the SCAs were first identified on 3D-time-of-flight MR-angiography (TOF-MRA) images and only then rated individually on DWI slices.^18^ The nature of the DWI signal in patients with GCA was also investigated in that study and concluded that it most likely represents a mixture of the so-called ‘T2 shine-through effect’ and a true diffusion restriction.^18^ Only in very large SCAs, it is possible to visualise a single wall with the typical resolution of DWI. In most instances, the signal from both walls blurs into a single ‘dot-shaped’ signal, which also includes partial volume effects.

If it was possible to identify vasculitis of SCAs using only DWI without the use of a TOF-MRA or other sequences, it may be possible to screen for GCA during image acquisition with the option to add a T1-BB on the fly. For this approach to work, a much simpler grading system would be needed, which could be completed in less than a minute. Also, an easy-to-use DWI assessment method would facilitate its application by clinicians who do not specialise in vasculitis imaging, such as emergency physicians or general rheumatologists. We, therefore, created a rating method based on ‘Gestalt’, a pattern recognition-based approach: the DWI-Scrolling-Artery-Sign (DSAS).^19–21^

In the present study, the diagnostic performance of the DSAS is assessed and compared to the T1-BB.^18^ To investigate whether the DSAS can also be used by less experienced readers, the diagnostic performance of a complete novice is compared with that of vasculitis imaging experts.

## Methods

This retrospective, monocentric study was conducted in accordance with the Declaration of Helsinki at the University Hospital Bern, Switzerland, a tertiary referral centre for vasculitis. It was approved by the Ethics Committee Bern, Switzerland, in 2021 (ID: 2021–02169); all participants gave their informed consent. The manuscript is written in accordance with the ‘Standards for Reporting of Diagnostic Accuracy Studies’ guidelines.^22^ The patient population (n=156) and image acquisition were identical to our previous publication on DWI in suspected GCA, where more information on patients, including detailed patients’ characteristics, the flow chart, and detailed DWI parameters can be found.^18^

### Study population

Inclusion criteria: age ≥50 years; evaluation for suspected GCA or suspected GCA relapse; MR scan of the head at the time of evaluation performed between 1 January 2018 and 31 December 2021; informed consent.^18^ Exclusion criteria: image artefacts precluding rating; missing DWI or T1-BB; non-GCA vasculitis; for relapses only—sustained complete clinical remission and normal C reactive protein for less than 4 months after treatment stop.^18^ From a total of 208 consecutive patients, which were retrospectively identified by screening hospital records, 52 patients were excluded (21 patients with GCA, 31 patients without GCA), including 35 patients due to a missing T1-BB-sequence, 2 with no DWI images, 4 with vasculitis other than GCA, 10 suspected GCA relapses and 1 with severe image artefacts.^18^ Of the 156 included patients, 59% were women and the median age was 71 years.^18^ The 69 patients without GCA had the following final diagnoses: 23 polymyalgia rheumatica, 10 primary headache, 9 non-arteritic anterior ischaemic optic neuropathy, 9 polyarthritis, 2 each with retinal artery occlusion, prominent TA, infection, lymphoma or sarcoidosis and 8 with other diagnoses.^18^ The clinical diagnosis ≥6 months after the initial diagnosis was used as diagnostic reference. It was determined independently by two senior rheumatologists (LS, PS or FL) based on all available medical records; the classification as GCA or non-GCA was identical for both experts.^18^ Relapses additionally had to meet the EULAR (European Alliance of Associations for Rheumatology) criteria for GCA relapses as defined by Hellmich *et al.* 5

### Image acquisition

All images were acquired on 3-Tesla scanners (Siemens, Germany) with 20-channel or 64-channel phased-array head and neck coils.^18^ Axial images were acquired between the hard palate and the vertex.^18^ Slice thickness was 0.5 mm for the TOF-MRA and 4–5 mm for DWI.^18^ DWI was a readout-segmented multishot echo-planar imaging sequence (Resolve) in 99%. 18 23 In more than 93% of patients, the following sequence parameters were used (b-value was 1000 s/mm^2^): acquisition matrix 192×192, field of view 220×220 mm, voxel size 1.15×1.15×4.0 mm, flip angle 180°; median of TR (repetition time) and TE (echo time) was 5750 ms and 61 ms, respectively.^18^ The post-contrast T1-BB sequence had a spatial resolution of approximately 0.195×0.260 mm; 30 slices with slice thickness of 3 mm and slice spacing of 6 mm were acquired with a TR of 500 ms, a TE of 22 ms, an acquisition matrix of 1’024×768 and a field of view of 200×200 mm.^18 24^

### Image evaluation

Readers were blinded to the reference diagnosis and all clinical information apart from age and sex; images were coded. Images were reread by LS (146 scans) and PS (30 scans), senior rheumatologists and vasculitis imaging experts with 12 and 11 years of work experience. Twenty MR scans were reread for inter-reader analysis between experts. The DWI images were examined for the presence of DSAS without any images from other sequences being visible on the screen. Segmental assessment of the DWI and T1-BB sequences was subsequently performed.^18^ All scans were also reread exclusively for the presence of the DSAS by SB, a medical school graduate who had never assessed MRI scans before. SB received a 20 min instruction by LS, which included an introduction to the software and a demonstration of five cases with and three without a positive DSAS.

### Rating of the arteries

**Figure 1:**
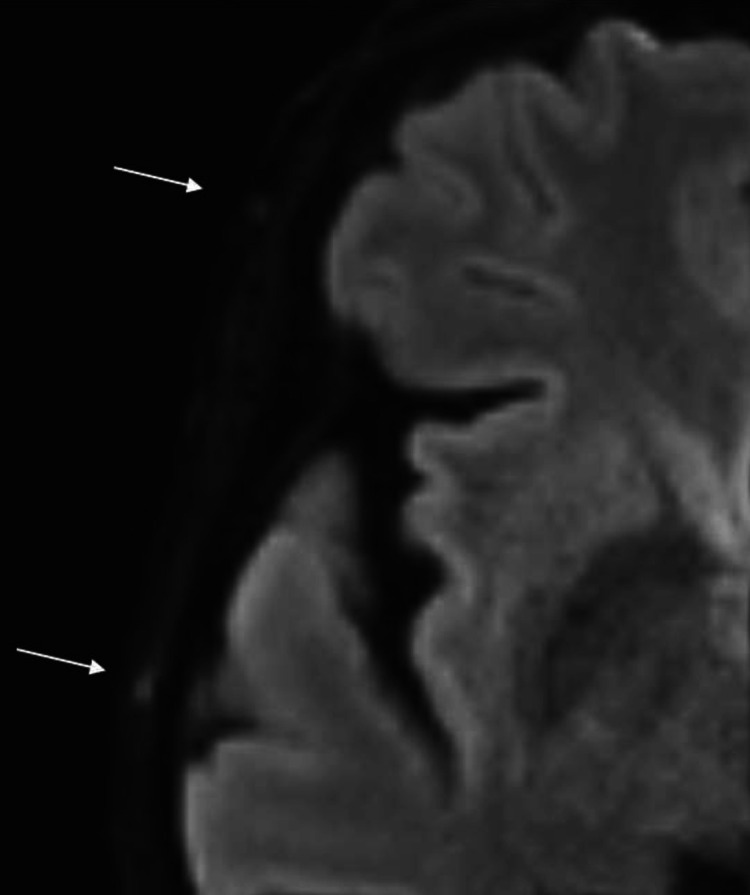
Pathological DWI signals. Example of a patient with histologically proven giant cell arteritis: The right frontal branch (upper arrow) shows a DWI-score of 2, the right parietal branch (lower arrow) a DWI-score of 3 (according to Seitz *et al*).^18^ DWI, diffusion-weighted imaging.

The spatial resolution of a typical DWI sequence is low (>1 mm) and normal SCAs are often not visible. It is not possible to know with certainty where the SCAs are located, which branch a signal belongs to or if the signal is from an artery. Thus, the DSAS cannot be assessed on a segment level without other sequences but instead was assessed for the following regions: frontotemporal and occipital, left and right. Definition of the DSAS: presence of hyperintense DWI signals in the subcutaneous tissue that gives the impression of a blood vessel when scrolling through a stack of images. Structures deeper than the subcutaneous tissue, that is, below the temporal fascia, for example, in muscle or bone, were not rated. A DWI signal was considered hyperintense, if it was consistent with a score of ≥2 according to the semiquantitative DWI grading scale for arterial segments defined by Seitz *et al*. 0=artery not visible; 1=artery slightly visible; 2=artery prominently visible; 3=artery brightly visible.^18^ Examples for DWI signals grades 2 and 3 are provided in figure 1. Slices were not counted, only the visual impression was considered. Online supplemental file 2 shows an example for a positive DSAS . Scans were considered to show vasculitis, if a DSAS was detected in ≥1 region. For the T1-BB, 10 segments were identified with the crosshair on corresponding TOF-MRA images: common superficial TA, frontal and parietal TA branches, the posterior auricular artery and the occipital artery. The TA segments were grouped into the frontotemporal regions, the posterior auricular and occipital arteries into the occipital regions. The T1-BB was rated according to Bley *et al*: 0, no mural thickening and no mural enhancement; 1, no mural thickening with slight mural enhancement; 2, mural thickening with prominent mural enhancement; 3, strong mural thickening with strong mural and perivascular enhancement.^24–27^ The scan was considered consistent with vasculitis, if ≥1 segment showed a T1-BB score of 2 or 3.^25^

10.1136/rmdopen-2023-003652.supp2 Supplementary video

### Statistics

Statistical analysis was performed using Stata (V.17.0), figures were made with R.^28 29^ For continuous variables, patient characteristics are reported as median with IQR, for categorical variables as absolute and relative frequencies. Comparison for continuous and categorical variables was made using the Mann-Whitney-Wilcoxon and Fisher’s exact test, respectively. Absolute and relative frequencies with Wilson 95% CI were used to report the proportion of correct classifications, sensitivity and specificity. Comparison of sensitivity and specificity between methods was made using the McNemar’s test. To quantify binary agreement at the patient or region level Cohen’s kappa with an analytical 95% CI was used. The area under the curve (AUC) of the receiver-operating characteristic (ROC) is reported with asymptotic DeLong 95% CI. Optimal cut points were determined using the methods from Liu and Youden.^30 31^

## Results

Results include data from 156 patients, 87 (55.8%) with GCA and 69 (44.2%) with other diagnoses. New-onset GCA was suspected in 151 (96.8%) patients and a GCA relapse in five (3.2%).^18^ Patients with suspected relapses presented 25 to 285 months after the initial diagnosis, all with cranial symptoms and elevated C reactive protein (34–123 mg/L). Twenty-eight (17.9%) patients showed non-cranial signs or symptoms only, 128 (82.1%) showed cranial manifestations (for example new-onset headache, scalp tenderness, jaw claudication, vision loss or thickening of temporal arteries). More detailed patients’ characteristics can be found elsewhere.^18^

Measures of diagnostic accuracy for DSAS for experts are presented in table 1. For the total population, a correct diagnosis was made with DSAS in 129/156 (82.7%, 95% CI 76.0 to 87.8%) patients, the sensitivity was 73.6% (95% CI 63.4 to 81.7%) and the specificity was 94.2% (95% CI 86.0 to 97.7%). For patients with cranial manifestations, a correct diagnosis was made with DSAS in 109/128 (85.2%, 95% CI 78.0 to 90.3%) patients, the sensitivity was 80.0% (95% CI 69.6 to 87.5%), the specificity was 92.5% (95% CI 82.1 to 97.0%). For both patient groups, sensitivity was significantly lower, and specificity tended to be higher for DSAS compared with T1-BB.^18^ More details on the proportion of correct diagnoses on the patient level for DSAS are shown in online supplemental table S1.

10.1136/rmdopen-2023-003652.supp1 Supplementary data

**Table 1:** Measures of diagnostic accuracy for the DSAS compared with the reference diagnosis (expert readers)

	Abnormal test— GCA	Sensitivity*	P value	Normal test— no GCA	Specificity*	P value	Positive LR ^†^	Negative LR ^†^	PPV ^*^	NPV ^*^	Correct diagnosis‡
Total study population (n=156)	64/87	73.6% (63.4–81.7%)	<0.001^§^	65/69	94.2% (86.0–97.7%)	0.16 ^§^	12.69 (4.86–33.12)	0.28 (0.20–0.40)	94.1% (85.6–98.4%)	73.9% (63.4–82.7%)	129 (82.7%, 76.0–87.8%)
Patients with cranial manifestations (n=128)	60/75	80.0% (69.6–87.5%)	0.002 ^§^	49/53	92.5% (82.1–97.0%)	0.65 ^§^	10.60 (4.10–27.38)	0.22 (0.14–0.34)	93.8% (84.8–98.3%)	76.6% (64.3–86.2%)	109 (85.2%, 78.0–90.3%)
Patients with GC prior to MRI (n=52)	21/28	75.0% (56.6–87.3%)	1.00 ^¶^	23/24	95.8% (79.8–99.3%)	1.00 ^¶^	18.00 (2.61–124.08)	0.26 (0.14–0.50)	95.5% (77.2–99.9%)	76.7% (57.7–90.1%)	44 (84.6%, 72.5–92.0%)
Patients without GC prior to MRI (n=104)	43/59	72.9% (60.4–82.6%)	n.a.	42/45	93.3% (82.1–97.7%)	n.a.	10.93 (3.62–32.98)	0.29 (0.19–0.44)	93.5% (82.1–98.6%)	72.4% (59.1–83.3%)	85 (81.7%, 73.2–88.0%)

*% (95% CI).

†Ratio (95% CI).

‡n (%, 95% Cl).

§Compared to T1-BB MRI sequence (measures of diagnostic accuracy for T1-BB were published previously^18^).

¶Compared to patient group without glucocorticoids prior to MRI.

DSAS, diffusion-weighted imaging scrolling artery sign; GC, glucocorticoids; GCA, giant cell arteritis; LR, likelihood ratio; n.a, not applicable; NPV, negative predictive value; PPV, positive predictive value; T1-BB, T1-black-blood.

There were only four non-GCA patients with a DSAS and none of these patients had a DSAS in more than two regions, whereas of the 87 GCA patients, 33 (37.9%) had a DSAS in three or four regions, 14 (16.1%) in two regions and 17 (19.5%) in one region. The DSAS was more frequently detected in the frontotemporal compared with the occipital regions (107 vs 86 regions). More detailed information about the DSAS on a regional level is shown in table 2. Agreement between DSAS and T1-BB on the patient level was observed in 135/156 (86.5%, 95% CI 80.3 to 91.0%) with a kappa of 0.73 (95% CI 0.63 to 0.84). Agreement between DSAS and T1-BB on the regional level was observed in 499/624 regions (80.0%, 95% CI 76.6 to 82.9%) with a kappa of 0.59 (95% CI 0.53 to 0.65), with higher agreements for the frontotemporal regions. In occipital regions, DSAS was detected in 68 of 144 pathological T1-BB regions (47.2%), the proportion was 103/148 (69.6%) for the frontotemporal regions. Online supplemental table S2 and S3 display the binary agreement between DSAS and T1-BB on patient and regional levels.

**Table 2:** Results for DSAS on a regional level (for total study population)

	Total ^*^ (N=156)	No GCA ^*^ (N=69)	GCA ^*^ (N=87)	P value ^†^
Number of regions with DSAS				<0.001 ^‡^
0	88 (56.4%)	65 (94.2%)	23 (26.4%)	n.a.
1	20 (12.8%)	3 (4.3%)	17 (19.5%)	n.a.
2	15 (9.6%)	1 (1.4%)	14 (16.1%)	n.a.
3	7 (4.5%)	0 (0.0%)	7 (8.0%)	n.a.
4	26 (16.7%)	0 (0.0%)	26 (29.9%)	n.a.
Regions with DSAS				
None	88 (56.4%)	65 (94.2%)	23 (26.4%)	<0.001
Fronto-temporal left	50 (32.1%)	1 (1.4%)	49 (56.3%)	<0.001
Fronto-temporal right	57 (36.5%)	3 (4.3%)	54 (62.1%)	<0.001
Occipital left	32 (20.5%)	1 (1.4%)	31 (35.6%)	<0.001
Occipital right	36 (23.1%)	0 (0.0%)	36 (41.4%)	<0.001

*N (%).

†P value for comparison of No GCA and GCA groups (Fisher’s exact test).

‡Because the number of regions is not independent, a p value is only calculated for the overall distribution.

DSAS, diffusion-weighted imaging scrolling artery sign; GCA, giant cell arteritis; n.a, not applicable.

**Figure 2:**
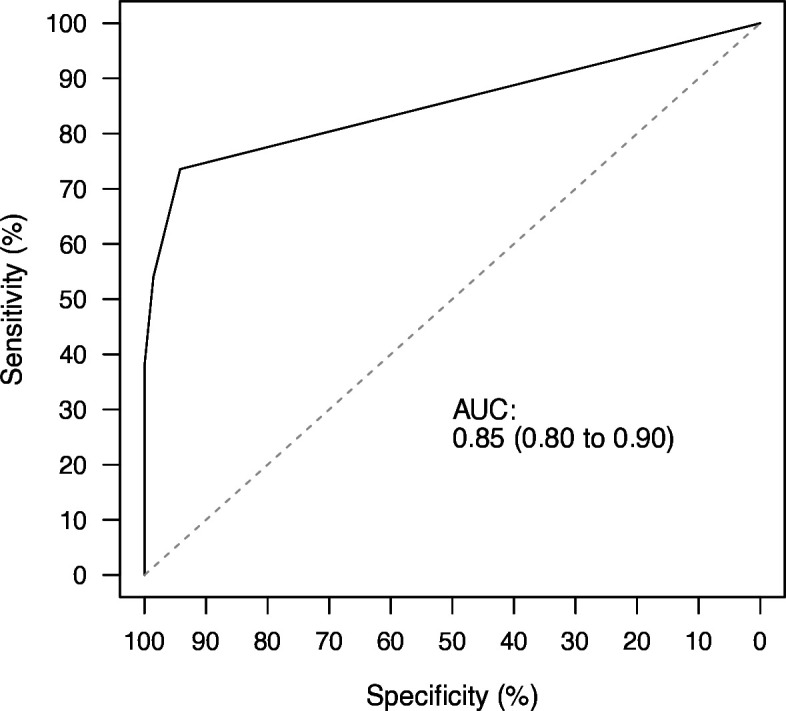
ROC curve for the number of regions per patient with a positive DSAS compared with the reference diagnosis: based on 624 regions from 156 patients. Area under the curve (AUC): 0.85 (95% CI 0.80 to 0.90). DSAS, diffusion-weighted imaging scrolling artery sign; ROC, receiver-operating characteristic.

Figure 2 shows the ROC curve for the number of regions per patient with a positive DSAS; the AUC was 0.85 (95% CI 0.80 to 0.90). Table 3 shows the measures of diagnostic accuracy at different cut-points for the number of regions with a positive DSAS per patient. The optimal cut-off was ≥1 as reported by Liu’s method and Youden’s index. The cut-off of ≥2 regions had a specificity of 98.6% (95% CI 92.2 to 99.7%) and a positive likelihood ratio of 37.28 (95% CI 5.28 to 263.40) for the diagnosis of GCA, sensitivity was 54.0% (95% CI 43.6 to 64.1%).

**Table 3:** Measures of diagnostic accuracy for number of regions with DSAS compared with reference diagnosis (expert readers; total study population)

Cut-point	Sensitivity ^*^	Specificity ^*^	Correctly classified ^*^	Positive LR ^†^	Negative LR†	PPV ^*^	NPV ^*^
≥ 1 region	73.6% (63.4–81.7%)	94.2% (86.0–97.7%)	82.7% (76.0–87.8%)	12.69 (4.86–33.12)	0.28 (0.20–0.40)	94.1% (85.6–98.4%)	73.9% (63.4–82.7%)
≥ 2 regions	54.0% (43.6–64.1%)	98.6% (92.2–99.7%)	73.7% (66.3–80.0%)	37.28 (5.28–263.40)	0.47 (0.37–0.59)	97.9% (88.9–99.9%)	63.0% (53.1–72.1%)
≥ 3 regions	37.9% (28.5–48.4%)	100% (94.7–100%)	65.4% (57.6–72.4%)	n.a.	0.62 (0.53–0.73)	100% (89.4–100%)	56.1% (46.9–65.0%)
≥ 4 regions	29.9% (21.3–40.2%)	100% (94.7–100%)	60.9% (53.1–68.2%)	n.a.	0.70 (0.61–0.80)	100% (86.8–100%)	53.1% (44.1–61.9%)

Optimal cut-point: Liu≥1 region; Youden≥1 region. Area under the curve: 0.85 (95% CI 0.80 to 0.90).

*% (95% CI).

† Ratio (95% CI).

DSAS, DWI-Scrolling-Artery-Sign; LR, likelihood ratio; n.a, not applicable; NPV, negative predictive value; PPV, positive predictive value.

Table 4 shows the measures of diagnostic accuracy for the DSAS for a novice. The correct diagnosis was given by the novice in 118/156 (75.6%, 95% CI 68.3 to 81.7%) patients in the total population and in 100/128 (78.1%, 95% CI 70.2 to 84.4%) patients with cranial manifestations. Compared with the DSAS read by experts, the sensitivity and the proportion of correct diagnosis was significantly lower for DSAS assessed by the novice for both patient groups, but the specificity was equally high (95.7%, 95% CI 88.0 to 98.5%, for the total patient population).

**Table 4:** Measures of diagnostic accuracy for the DSAS compared with the reference diagnosis for the novice reader

	Abnormal test—GCA	Sensitivity ^*^	P value (vs expert)	Normal test—No GCA	Specificity ^*^	P value (vs expert)	Correct diagnosis ^†^	P value (vs expert)
Total study population (n=156)	52/87	59.8% (49.3–69.4%)	0.003	66/69	95.7% (88.0–98.5%)	0.56	118 (75.6%, 68.3–81.7%)	0.003
Patients with cranial manifestations (n=128)	50/75	66.7% (55.4–76.3%)	0.008	50/53	94.3% (84.6–98.1%)	0.56	100 (78.1%, 70.2–84.4%)	0.008

*% (95% CI).

†N (%, 95% CI).

DSAS, diffusion-weighted imaging scrolling artery sign; GCA, giant cell arteritis; LR, likelihood ratio.

The results of 20 patients were analysed for an inter-rater analysis between experts. The correct diagnosis with DSAS was given by one expert in 19 (95.0%, 95% CI 76.4 to 99.1%) and by the second expert in 18 (90.0%, 95% CI 69.9 to 97.2%) patients with agreement in 19 patients (95.0%; 95% CI 76.4 to 99.1%) and a kappa of 0.90 (95% CI 0.70 to 1.00). Binary agreement was present in 73/80 (91.3%, 95% CI 83.0 to 95.7%) regions, with a kappa of 0.81 (95% CI 0.68 to 0.94); agreement was slightly higher for the frontotemporal regions. More details on the proportion of correct diagnoses as well as on the binary agreement between experts are presented in online supplemental tables S4 and S5.

Results from all 156 patients were available for inter-rater analysis between experts and the novice. Agreement was observed for 137/156 (87.8%, 95% CI 81.8 to 92.1%) patients, with a kappa of 0.75 (95% CI 0.64 to 0.85). On the regional level, agreement was present in 569/624 (91.2%, 95% CI 88.7 to 93.2%) regions with a kappa of 0.77 (95% CI 0.71 to 0.83). Online supplemental tables S6 and S7 show more details on agreement between experts and the novice for the DSAS on a patient and regional level.

A subanalysis for patients with and without GC treatment at the time of the MRI was additionally performed. For the 52/156 patients (33.3%) who received GC prior to the MRI, the median duration of GC therapy was 4 days (IQR 1–118 days, range 1–5755 days; one missing value) and the median oral prednisone equivalent dose was 60 mg (IQR 14–625 mg; range 1–1250 mg; one missing value). Of the 24 patients treated with GC for more than 1 week, 10 received prednisone doses of ≤7.5 mg/day. Of the 52 patients with GC pre-treatment, 45 (86.5%) presented with cranial manifestations. No relevant difference of measures of diagnostic accuracy was detectable between the patients with and without GC pretreatment (table 1).

## Discussion

The present study investigated a very simple DWI assessment method for suspected GCA of the SCAs, the DSAS, which can be performed in about 30–45 s and is based on a pattern recognition approach for image analysis.^19–21^ The results show that this novel approach has a high diagnostic accuracy, even if performed by a non-expert. In the previous study by Seitz *et al* on the use of DWI in suspected GCA, a segmental and slice-by-slice assessment of the DWI was used with prior identification of arteries on TOF-MRA.^18^ The presence of pathological DWI signals on at least two slices proved to be the most accurate DWI rating method for suspected GCA.^18^ With 10 segments to be examined on several layers, the time for the segmental rating in the previous study was about 4 min, which was similar for the T1-BB sequence.^18^

Surprisingly, the DSAS showed an almost identical diagnostic accuracy for the diagnosis of GCA compared with the segmental DWI rating method reported by Seitz e*t al* (specificity 94.2%, sensitivity 75.9%), even though no other MRI sequence was used, the assessment was performed in regions instead of segments and the rating time was approximately 80% less.^18^ As for the segmental DWI rating method, compared to the results from the T1-BB method reported by Seitz *et al*, the specificity of the DSAS was numerically higher (94.2% vs 88.4%) and the sensitivity was significantly lower (73.6% vs 88.5%) for the total population.^18^ The diagnostic accuracy of the DSAS matches that of the T1-BB in the largest prior study with the same reference standard (sensitivity 78.4%, specificity 90.4%).^27^ It is also very similar to the pooled sensitivity (82%) and specificity (92%) of the T1-BB reported by the systematic literature review informing the 2023 update of the EULAR recommendations for imaging in large-vessel vasculitis.^6 32^ Differences in patient selection to other studies examining T1-BB in suspected GCA were discussed in detail elsewhere.^18^

The ROC curve analysis confirmed a DSAS in ≥1 region as the optimal cut-off. With a cut-off of a DSAS in ≥2 regions, the specificity was 98.6%, allowing a diagnosis with a very high level of certainty in more than half of the patients with GCA (47/87, 54 %). For a cut-off of a DSAS in ≥3 regions, specificity was 100%, which makes it very unlikely to find patients without GCA with this constellation.

**Figure 3:**
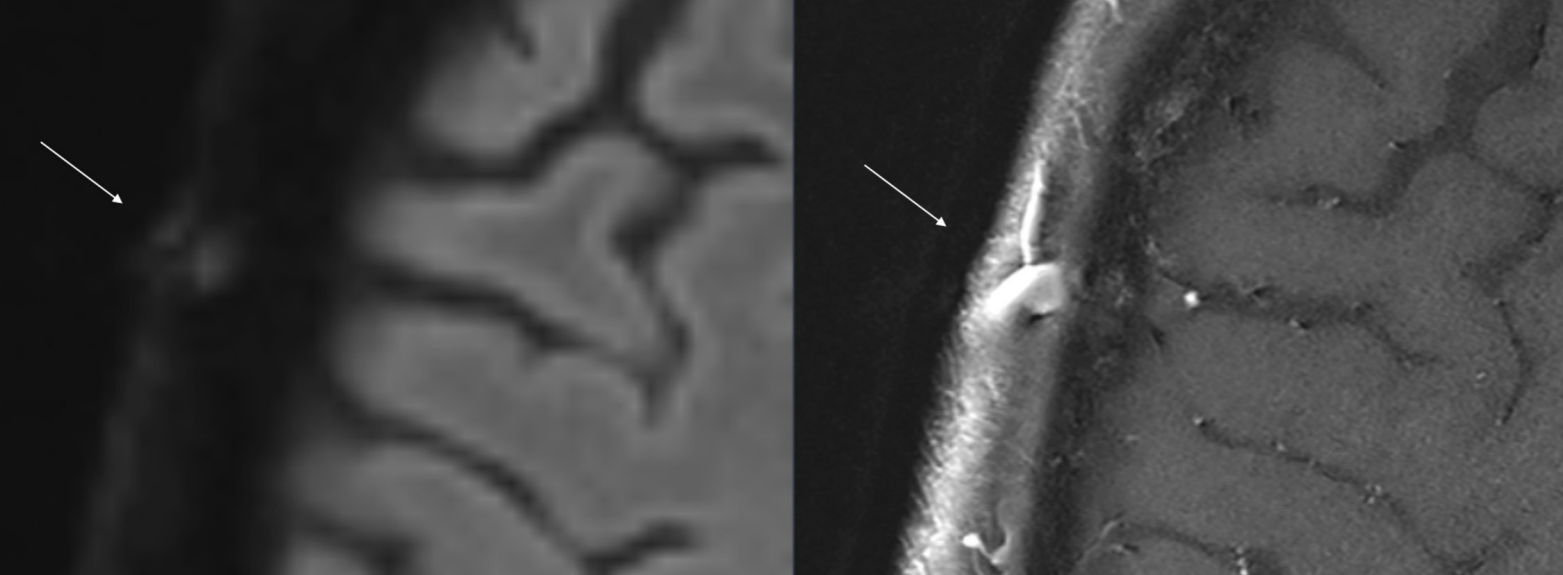
Vein of the scalp with false-positive DSAS: a large vein in the right parietal region shows a DWI-score of 2–3 (left panel). The right panel shows the corresponding T1-black-blood image, where the vein has the typical hyperintense lumen with a large diameter in contrast to the black and smaller lumen of arteries. DSAS, DWI scrolling artery sign; DWI, diffusion-weighted imaging.

Only 5/624 (0.8 %) of the regions showed a false-positive DSAS, with only one patient having two false-positive regions. Three out of five false-positive DSAS were identified as DWI-positive veins. Large veins, especially with low flow, can rarely exhibit an identical DWI signal as arterial segments with vasculitis (figure 3).^18^ Checking on a TOF-MRA whether a positive DSAS is corresponding to an artery would quickly resolve this problem. To minimise the risk of inadvertently assessing a vein, it is crucial to assess only the epifascial subcutaneous layer for the presence of the DSAS. Agreement between DSAS and T1-BB was better on the patient level compared with the regional level; 21/156 (13.5%) patients and 125/624 (20%) regions were assessed differently. The DSAS may miss an affected segment in one region but still detects vasculitis in another. This leads to a better performance on the patient level. Similar to the segmental DWI rating method, DSAS mainly detects clearly altered arteries and tends to miss very small or only slightly affected arteries, which may explain the higher specificity compared with T1-BB.^18^ The binary agreement was the lowest in the occipital regions. DSAS detected only 47.2% of T1-BB-positive occipital and 71.5% of T1-BB-positive frontotemporal regions. Rating of the DWI is particularly challenging in the occipital regions due to non-arterial structures such as occipital lymph nodes showing similar findings.^18^ Without the aid of TOF-MRA or crosshairs, it is difficult to distinguish these structures from an arterial signal, which is often immediately adjacent. However, as the occipital artery is rarely affected in isolation, a lower sensitivity for the occipital region is unlikely to have a relevant impact on the diagnostic performance.^33^ This was confirmed in our previous study, where the analysis restricted to frontal and parietal branches showed a nearly identical diagnostic performance.^18^

Although the novice had no previous experience, the DSAS still performed very well, with the same specificity but significantly lower sensitivity compared with experts. In clinical practice, we expect a better performance if the DSAS is rated by junior physicians or radiographers, who are familiar with the assessment of MRI scans. Although the difference in experience was maximised and the training minimised, the level of agreement between experts and novice was still substantial. We expect it to be even better with a dedicated training programme. The positive effects of a formal training were previously demonstrated for ultrasound in suspected GCA.^34^ We are not aware of any studies that have investigated agreement between experts and non-experts or the effect of training on MRI reporting in suspected GCA.

The inter-rater analysis both on the patient and region level showed an almost perfect agreement for the DSAS between experts.^35 36^ The inter-rater agreement of DSAS is comparable to the segmental DWI rating method and appears to be higher than that of T1-BB, which underlines the practicality of the method. 18 27 35–37

Prior studies found GC therapy having a major influence on diagnostic accuracy of diagnostic imaging in suspected GCA, not only for FDG-PET but also for cranial MRI.^38 39^ We investigated this by comparing measures of diagnostic accuracy between groups with and without GC pretreatment. No relevant difference could be detected. The most likely explanations are the very short duration of GC treatment (median 4 days), relatively low prednisone doses in many patients with a treatment duration of more than 1 week, and the fact that 86.5% of patients with GC treatment had cranial manifestations at the time of imaging (this subgroup had higher sensitivity than the total study population (80.0 vs 73.6%).

The study has several limitations. The main problem with the retrospective design is the potential for selection bias. Although we took great care to include every available patient in our centre it cannot be excluded, that some patients were missed and that this affected the results. Our study population had a high proportion of cases with GCA, which may have been related to the fact that the study was conducted at a vasculitis referral centre and that 35 patients with missing T1-BB images were excluded, a situation that was more common in patients without GCA (24/35).^18^ However, large prospective imaging studies in GCA had similarly high proportions of patients with GCA.^27 37^ Because we did not exclude patients with prior glucocorticoid treatment, or if they presented with suspected relapses, we included a broader population than previously published prospective MRI studies for suspected GCA to simulate a real-life situation.^18 24–27 37^ While a potential inclusion bias is relevant for comparing measures of diagnostic accuracy with other studies, it is not relevant for intraindividual comparison of different MR sequences and scoring schemes. Because experts evaluated the patients’ electronic medical records to arrive at the reference diagnosis, blinding to the T1-BB results of the MRI scans, as documented in the patient charts, was not possible. However, multiple diagnostic tests are usually performed in our centre when GCA is suspected: ultrasonography of the SCAs, axillary and subclavian arteries, and/or TA biopsy, and/or FDG-PET-CT were performed in 155/156 (99.4%) patients. Compared with the expert diagnosis, the T1-BB results documented in the patient charts did not match in 29 (18.6%) patients. This suggests that the experts did not rely on T1-BB MRI results in the patients’ records for their diagnosis. It is essential to note that experts were blinded to results of the MRI reread. Also, DWI imaging was not rated in the past and DWI results of the SCAs were not included on any of the medical records. Thus, while a certain influence of the T1-BB MRI results, as documented in the charts, cannot be completely excluded, DWI results did not influence the expert diagnosis in any way. This makes circular reasoning with respect to the DSAS and the experts’ reference diagnosis highly unlikely.

It remains unknown, if the diagnostic performance of the DSAS is similar on scanners from other manufacturers and if less advanced DWI sequences are used. In everyday clinical practice, we regularly assess MRI images from scanners from different manufacturers, with the DSAS also visible on these images. However, these scans have not been systematically assessed. In addition, it would have been ideal to have all MRIs assessed by two experts. Due to limited resources, this was not possible.

The role for monitoring of vascular inflammation in GCA with imaging is still unclear as the significance of ongoing vessel wall enhancement remains unknown.^6^ Recent EULAR recommendations do not recommend imaging for GCA in clinical and biochemical remission, but state that imaging may be used in case of suspected relapse.^6^ As such, we believe it to be important to gain knowledge about imaging in this scenario as well, which is why we included patients with suspected relapse in whom imaging would be considered to be performed in clinical practice. Example images of complete normalisation in clinical remission and return of pathological DWI signals in case of GCA relapse are available elsewhere.^18^

Due to the lower spatial resolution, DWI and DSAS were expected to have a lower sensitivity than the T1-BB sequence for GCA, but their specificity may even be higher.^18^ In addition, an excellent inter-rater agreement was found for the DSAS. The DWI sequence was acquired in approximately two min and does not require contrast agents. Together with an extremely short rating time and a very high specificity of the DSAS even for complete novices, this opens novel opportunities for potential clinical use. In certain clinical scenarios, such as headache in a patient >50 years of age, radiologists or possibly even radiographers could routinely assess the DSAS while the patient is still in the scanner, even if GCA was not initially suspected. This would be easy to implement as DWI is usually the first sequence of an MRI protocol of the head. In cases with a DSAS in ≥2 regions, additional imaging may not necessarily be required. The spontaneous addition of a T1-BB at the end of the protocol could be an option in patients with a DSAS in one region. This could reduce the time to diagnosis and possibly costs, as in our experience, these MRI scans are often considered normal because SCAs are not routinely assessed with DWI.

In daily practice, for many centres and clinicians, it is often a challenge to obtain specialised imaging quickly enough when GCA is suspected, especially because sensitivity often decreases rapidly with glucocorticoid therapy. Experienced sonographers or an appointment for an MRI scan including a T1-BB sequence may not be available in time or not at all. Given the very good diagnostic performance of DWI in suspected GCA, a very short MRI protocol may be an option in such situations. Such a protocol, including a DWI sequence with a high spatial resolution and a TOF-MRA, may require less than five min of scanning time. Such a fast-track approach would likely fit into an already busy outpatient radiology or emergency department schedule and allow for a same-day appointment.

Prospective studies are needed to determine the definitive value of DWI and the DSAS for the diagnosis of GCA. Because DWI is available on every MRI scanner worldwide, the DSAS can be implemented immediately into clinical practice.

## Data Availability

Data are available upon reasonable request.
