# Supplementary material for: DWI scrolling artery sign for the diagnosis of giant cell arteritis: a pattern recognition approach
Source: RMD Open. 2024 Mar 22;10(1):e003652. doi: 10.1136/rmdopen-2023-003652 (PMC10961581; doi:10.1136/rmdopen-2023-003652)
Supplement: Supplementary data [file rmdopen-2023-003652supp001.pdf]

Supplemental material

"The DWI scrolling artery sign for the diagnosis of giant cell arteritis - a pattern recognition approach"

Supplemental Tables S1 – S7

Supplemental Video S1

Seitz L, *et al. RMD Open* 2024; 10:e003652. doi: 10.1136/rmdopen-2023-003652

Supplemental Table S1. Correct diagnosis of DSAS compared to the reference diagnosis on a patient level

Total study population

	**Total** [a] *(N = 156)*	**No GCA** [a] *(N = 69)*	**GCA** [a] *(N = 87)*	**Correct diagnosis** [a] *(N = 156)*
**DSAS**				129 (82.7%, 76.0 to 87.8%)
No Vasculitis	88 (56.4%, 48.6 – 63.9%)	65 (94.2%, 86.0 - 97.7%)	23 (26.4%, 18.3 - 36.6%)	
Vasculitis	68 (43.6%, 36.1 - 51.4%)	4 (5.8%, 2.3 - 14.0%)	64 (73.6%, 63.4 - 81.7%)	

Patients with cranial manifestations

	**Total** [a] *(N = 128)*	**No GCA** [a] *(N = 53)*	**GCA** [a] *(N = 75)*	**Correct diagnosis** [a] *(N = 128)*
**DSAS**				109 (85.2%, 78.0 - 90.3%)
No Vasculitis	64 (50.0%, 41.5 - 58.5%)	49 (92.5%, 82.1 - 97.0%)	15 (20.0%, 12.5 - 30.4%)	
Vasculitis	64 (50.0%, 41.5 - 58.5%)	4 (7.5%, 3.0 - 17.9%)	60 (80.0%, 69.6 - 87.5%)	

[a], n (%, 95% confidence interval). DSAS, diffusion-weighted imaging scrolling artery sign.

Supplemental Table S2: Binary agreement between DSAS and T1-BB on the patient level (total population)

	**T1-BB: No Vasculitis** [a]	**T1-BB: Vasculitis** [a]	**Agreement** [a]	**Cohen's kappa** [b]
**DSAS**			135 (86.5%, 80.3 – 91.0%)	0.73 (0.63 – 0.84)
No Vasculitis	69 (97.2%, 90.3 – 99.2%)	19 (22.4%, 14.8 – 32.3%)		
Vasculitis	2 (2.8%, 0.8 – 9.7%)	66 (77.6%, 67.7 – 85.2%)		

[a], n (%, 95% confidence interval); [b], kappa (95% confidence interval). DSAS, diffusion-weighted imaging scrolling artery sign; T1-BB, T1-black-blood.

Supplemental Table S3: Binary agreement between DSAS with T1-BB on the regional level (total population)

	**T1-BB: No Vasculitis** [a]	**T1-BB: Vasculitis** [a]	**Agreement** [a]	**Cohen's kappa** [b]
**Fronto-temporal left**			132 (84.6%, 78.1 – 89.4%)	0.68 (0.57 - 0.79)
Normal	84 (97.7%, 91.9 - 99.4%)	22 (31.4%, 21.8 - 43.0%)		
Pathological	2 (2.3%, 0.6 – 8.1%)	48 (68.6%, 57.0 - 78.2%)		
**Fronto-temporal right**			131 (84.0%, 77.4 - 88.9%)	0.68 (0.57 - 0.79)
Normal	76 (97.4%, 91.1 - 99.3%)	23 (29.5%, 20.5 - 40.4%)		
Pathological	2 (2.6%, 0.7 – 8.9%)	55 (70.5%, 59.6 - 79.5%)		
**Occipital left**			120 (76.9%, 69.7 – 82.8%)	0.50 (0.38 - 0.62)
Normal	88 (100%, 95.8 - 100%)	36 (52.9%, 41.2 - 64.3%)		
Pathological	0 (0.0%, 0.0 – 4.2%)	32 (47.1%, 35.7 – 58.8%)		
**Occipital right**			116 (74.4%, 67.0 – 80.6%)	0.48 (0.36 - 0.60)
Normal	80 (100%, 95.4 - 100%)	40 (52.6%, 41.6 - 63.5%)		
Pathological	0 (0.0%, 0.0 – 4.6%)	36 (47.4%, 36.5 - 58.4%)		
**Overall**			499 (80.0%, 76.6 – 82.9%)	0.59 (0.53 - 0.65)
Normal	328 (98.8%, 96.9 - 99.5%)	121 (41.4%, 35.9 - 47.2%)		
Pathological	4 (1.2%, 0.5 - 3.1%)	171 (58.6%, 52.8 - 64.1%)		

[a], n (%, 95% confidence interval); [b], kappa (95% confidence interval). DSAS, diffusion-weighted imaging scrolling artery sign; T1-BB, T1-black-blood

Seitz L, *et al. RMD Open* 2024; 10:e003652. doi: 10.1136/rmdopen-2023-003652

Supplemental Table S4: Correct diagnosis for two expert readers for DSAS compared to the reference diagnosis for a subset of 20 patients

	Total [a] *(N = 20)*	No GCA [a] *(N = 8)*	GCA [a] *(N = 12)*	Correct diagnosis [a] *(N = 20)*
**Reader 1**				19 (95.0%, 76.4 - 99.1%)
No Vasculitis	9 (45.0%, 25.8 – 65.8%)	8 (100%, 67.6 – 100%)	1 (8.3%, 1.5 – 35.4%)	
Vasculitis	11 (55.0%, 34.2 - 74.2%)	0 (0.0%, 0.0 - 32.4%)	11 (91.7%, 64.6 – 98.5%)	
**Reader 2**				18 (90.0%, 69.9 - 97.2%)
No Vasculitis	8 (40.0%, 21.9 - 61.3%)	7 (87.5%, 52.9 - 97.8%)	1 (8.3%, 1.5 - 35.4%)	
Vasculitis	12 (60.0%, 38.7 - 78.1%)	1 (12.5%, 2.2 - 47.1%)	11 (91.7%, 64.6 – 98.5%)	

[a], n (%, 95% confidence interval). DSAS, diffusion-weighted imaging scrolling artery sign.

Supplemental Table S5. Binary agreement between two expert readers for DSAS for a subset of 20 patients

	Reader 2 – No Vasculitis [a]	Reader 2 – Vasculitis [a]	Agreement [a]	Cohen's kappa [b]
**Patient level (Reader 1)**				
**DSAS**			19 (95.0%, 76.4 – 99.1%)	0.90 (0.70 – 1.00)
No Vasculitis	8 (100%, 67.6 - 100%)	1 (8.3%, 1.5 – 35.4%)		
Vasculitis	0 (0.0%, 0.0 – 32.4%)	11 (91.7%, 64.6 – 98.5%)		
**Region level (Reader 1)**				
**Fronto-temporal left**			18 (90.0%, 69.9 - 97.2%)	0.79 (0.53 - 1.00)
Normal	11 (100%, 74.1 - 100%)	2 (22.2%, 6.3 - 54.7%)		
Pathological	0 (0.0%, 0.0 - 25.9%)	7 (77.8%, 45.3 - 93.7%)		
**Fronto-temporal right**			20 (100%, 83.9 - 100%)	1.00 (1.00 - 1.00)
Normal	10 (100%, 72.2 - 100%)	0 (0.0%, 0.0 - 27.8%)		
Pathological	0 (0.0%, 0.0 - 27.8%)	10 (100%, 72.2 - 100%)		
**Occipital left**			18 (90.0%, 69.9 - 97.2%)	0.74 (0.40 - 1.00)
Normal	14 (100%, 78.5 - 100%)	2 (33.3%, 9.7 - 70.0%)		
Pathological	0 (0.0%, 0.0 to 21.5%)	4 (66.7%, 30.0 - 90.3%)		
**Occipital right**			17 (85.0%, 64.0 - 94.8%)	0.66 (0.31 - 1.00)
Normal	12 (92.3%, 66.7 - 98.6%)	2 (28.6%, 8.2 - 64.1%)		
Pathological	1 (7.7%, 1.4 - 33.3%)	5 (71.4%, 35.9 - 91.8%)		
**Overall**			73 (91.3%, 83.0 - 95.7%)	0.81 (0.68 - 0.94)
Normal	47 (97.9%, 89.1 - 99.6%)	6 (18.8%, 8.9 - 35.3%)		
Pathological	1 (2.1%, 0.4 - 10.9%)	26 (81.3%, 64.7 - 91.1%)		

[a], n (%, 95% confidence interval); [b], kappa (95% confidence interval). DSAS, diffusion-weighted imaging scrolling artery sign.

Seitz L, *et al. RMD Open* 2024; 10:e003652. doi: 10.1136/rmdopen-2023-003652

Supplemental Table S6. Binary agreement between vasculitis experts and novice for DSAS on the patient level

	Novice – No Vasculitis [a]	Novice – Vasculitis [a]	Agreement [a]	Cohen's kappa [b]
**Vasculitis experts**				
**Total study population** *(n = 156)*			137 (87.8%, 81.8 –92.1%)	0.75 (0.64 – 0.85)
No Vasculitis	85 (84.2%, 75.8 – 90.0%)	3 (5.5%, 1.9 – 14.9%)		
Vasculitis	16 (15.8%, 10.0 – 24.2%)	52 (94.5%, 85.1 – 98.1%)		
**Patients with cranial manifestation** *(n = 128)*			111 (86.7%, 79.8 - 91.5%)	0.73 (0.62 – 0.85)
No Vasculitis	61 (81.3%, 71.1 – 88.5%)	3 (5.7%, 1.9 – 15.4%)		
Vasculitis	14 (18.7%, 11.5 - 28.9%)	50 (94.3%, 84.6 – 98.1%)		

[a], n (%, 95% confidence interval); [b], kappa (95% confidence interval); DSAS, diffusion-weighted imaging scrolling artery sign.

Supplemental Table S7. Binary agreement between vasculitis experts and novice for DSAS on the regional level

	Novice – Normal [a]	Novice – Pathological [a]	Agreement [a]	Cohen's kappa [b]
**Vasculitis experts**				
**Fronto-temporal left**			144 (92.3%, 87.0 - 95.5%)	0.81 (0.71 - 0.91)
Normal	105 (90.5%, 83.8 - 94.6%)	1 (2.5%, 0.4 - 12.9%)		
Pathological	11 (9.5%, 5.4 - 16.2%)	39 (97.5%, 87.1 - 99.6%)		
**Fronto-temporal right**			140 (89.7%, 84.0 - 93.6%)	0.78 (0.67 - 0.88)
Normal	93 (90.3%, 83.0 - 94.6%)	6 (11.3%, 5.3 - 22.6%)		
Pathological	10 (9.7%, 5.4 - 17.0%)	47 (88.7%, 77.4 - 94.7%)		
**Occipital left**			142 (91.0%, 85.5 - 94.6%)	0.72 (0.58 - 0.86)
Normal	118 (93.7%, 88.0 - 96.7%)	6 (20.0%, 9.5 - 37.3%)		
Pathological	8 (6.3%, 3.3 - 12.0%)	24 (80.0%, 62.7 - 90.5%)		
**Occipital right**			143 (91.7%, 86.3 - 95.1%)	0.74 (0.61 - 0.87)
Normal	118 (91.5%, 85.4 - 95.2%)	2 (7.4%, 2.1 - 23.4%)		
Pathological	11 (8.5%, 4.8 - 14.6%)	25 (92.6%, 76.6 - 97.9%)		
**Overall**			569 (91.2%, 88.7 - 93.2%)	0.77 (0.71 - 0.83)
Normal	434 (91.6%, 88.7 - 93.7%)	15 (10.0%, 6.2 - 15.8%)		
Pathological	40 (8.4%, 6.3 - 11.3%)	135 (90.0%, 84.2 - 93.8%)		

[a], n (%, 95% confidence interval); [b], kappa (95% confidence interval). DSAS, diffusion-weighted imaging scrolling artery sign.

Seitz L, *et al. RMD Open* 2024; 10:e003652. doi: 10.1136/rmdopen-2023-003652

Supplemental Video S1

A DWI scrolling artery sign (DSAS) in the right fronto-temporal region is shown (arrows). The video is displayed at four frames per second. The progress bar can be manually dragged back and forth to simulate scrolling through a stack of images.
